# Research capacity development in podiatry: the contribution of the Society of Chiropodists and Podiatrists

**DOI:** 10.1186/1757-1146-3-S1-P3

**Published:** 2010-12-20

**Authors:** Jackie Campbell

**Affiliations:** 1 Society of Chiropodists and Podiatrists, London, UK

## Objectives and relationship to conference themes

The development of a research culture within the Society of Chiropodists and Podiatrists, and the research capacity building within the profession that must underpin this, is an essential element of both the ‘learn’ and ‘achieve’ themes of the conference.

The role of professional bodies in developing professional practice and the maintenance of standards is unquestioned and is the main focus for the work of these bodies. However, research evidence is required to provide the evidence base for that professional practice. This need for evidence is becoming more urgent as, for example, funders of health-care services and fee-paying patients are increasingly requiring evidence of efficacy and effectiveness of interventions. In the UK, the move to commissioning of services for the National Health Service is accelerating this trend.

Professional bodies should therefore have a role in developing that evidence base as a core part of their business. However, particularly for the smaller allied health professions, scarce resources and other competing priorities mitigate against this. This poster will look at how the Society of Chiropodists and Podiatrists have begun to tackle this problem and will illustrate how, in less than five years, they have moved from research being a special interest of a few, to it being a core part of the organisation, with major successes for podiatrists in prestigious, competitive national research schemes.

## Relevance and impact of this topic

An evidence base for the profession is essential if it is to survive. This poster describes how that evidence base is being encouraged and developed within the professional body.

## Participant outcomes

Participants will gain an understanding and appreciation of the role of professional bodies in general, and the UK Society of Chiropodists and Podiatrists in particular, in developing the capacity to provide the evidence base for the profession.

